# Predicting Early and Late Readmissions Following Cytoreductive Surgery and Hyperthermic Intraperitoneal Chemotherapy

**DOI:** 10.1245/s10434-021-10414-2

**Published:** 2021-07-24

**Authors:** Eui Whan Moon, Jolene Si Min Wong, Amanda Hui Min See, Whee Sze Ong, Chee Ann Tan, Chin-Ann Johnny Ong, Claramae Shulyn Chia, Khee Chee Soo, Melissa Ching Ching Teo, Grace Hwei Ching Tan

**Affiliations:** 1 Division of Surgery and Surgical Oncology, Department of Sarcoma, Peritoneal and Rare Tumours (SPRinT), National Cancer Centre Singapore, Singapore, Singapore; 2 Department of Clinical Trials and Epidemiological Sciences, National Cancer Centre Singapore, Singapore, Singapore; 3 Laboratory of Applied Human Genetics, Division of Medical Sciences, National Cancer Centre Singapore, Singapore, Singapore; 4 SingHealth Duke-NUS Oncology Academic Clinical Program, Duke-NUS Medical School, Singapore, Singapore; 5 Institute of Molecular and Cell Biology, A*STAR Research Entities, Singapore, Singapore

## Abstract

**Background:**

Postoperative readmissions not only burden the healthcare system but may also affect clinical outcomes of cancer patients. Despite this, little is known about readmissions after cytoreductive surgery (CRS) and hyperthermic intraperitoneal chemotherapy (HIPEC), or their impact on survival outcomes.

**Patients and Methods:**

A single-institution retrospective cohort study of CRS-HIPEC procedures from April 2001 and September 2019 was performed. Early readmission (ERA) was defined as hospitalization within 30 days of discharge post-CRS/HIPEC, while late readmission (LRA) was defined as hospitalization between day 31 and 90 after discharge. Patient demographic, oncological, and perioperative factors were analyzed to identify predictors of readmission, and comparison of survival outcomes was performed.

**Results:**

Overall, 342 patients who underwent CRS-HIPEC were included in the study. The incidence of ERA and LRA was 18.5% and 7.4%, respectively. High-grade postoperative complication was the only independent predictor of ERA (HR 3.64, 95% CI 1.47–9.02), while comorbid hypertension (HR 2.71, 95% CI 1.17–6.28) and stoma creation (HR 2.83, 95% CI 1.23–6.50) were independent predictors for LRA. Patients with readmission had significantly worse disease-free survival than patients who had no readmission (NRA) (LRA 1.1 years, ERA 1.2 years, NRA 1.8 years, *p* = 0.002), and patients with LRA had worse median overall survival (2.1 years) than ERA patients (3.3 years) or patients without readmission (4.4 years) (*p* < 0.001).

**Conclusions:**

Readmission following CRS-HIPEC is associated with adverse survival outcomes. In particular, LRA may portend worse prognosis than ERA.

Over the past two decades, cytoreductive surgery (CRS) and hyperthermic intraperitoneal chemotherapy (HIPEC) have gradually gained acceptance as a treatment option for selected patients with peritoneal surface-based malignancies secondary to gastrointestinal or gynecological primaries.^1^–^3^ Complete cytoreduction during CRS is one of the most important predictors of survival outcomes.^4^ Therefore, it is common for multivisceral resection to be performed during CRS to ensure eradication of all macroscopic disease.^5^ As a result, reported rates of postoperative morbidity following CRS-HIPEC range from 10 to 50%, and mortality from 1 to 6%.^6^,^7^

In addition to morbidity and mortality, another postoperative metric attracting interest in the literature is postoperative readmission (RA) rates. Hospital readmissions after cancer-related surgeries not only contribute to higher costs of care but may also be associated with poorer clinical outcomes. For colon cancer-related colectomies, 1-year mortality for patients with 30-day RA versus without RA was reported to be 16% versus 7%, respectively,^8^ and similar trends were reported for patients with 90-day RA after surgery for bladder, esophageal, lung, and pancreatic cancers.^9^ Thirty-day RA rates post CRS-HIPEC have been reported to be between 11 and 24%, indications for which include digestive complications, pain, infection, and venous thromboembolism.^10^–^13^ Late RA occurring up to postoperative day 90 has been reported to occur at rates of up to 7.8–21%.^14^,^15^ Postoperative RA in patients who have undergone CRS-HIPEC not only poses a heavy financial burden on the healthcare system, but may also have significant implications for survival outcomes, as suggested by existing data on RA after other oncological surgeries.

Despite this, there is a paucity of data on predictors for hospital RA after CRS-HIPEC among the Asian population, and even fewer studies that evaluate its association with survival rates. To address these knowledge gaps, the aims of this study are to identify risk factors associated with early and late RA post CRS-HIPEC and their impact on oncologic outcomes.

## Patients and Methods

Ethical approval from the SingHealth Centralised Institutional Review Board was obtained for the conduct of this retrospective cohort study. Data were retrieved from a prospectively maintained database of patients who had undergone CRS-HIPEC at National Cancer Centre Singapore.

### Patient Selection

Patients were selected for CRS-HIPEC upon review and recommendation by a multidisciplinary tumor board discussion. All patients selected had Eastern Cooperative Group (ECOG) performance status of either 0 or 1 and no distant metastases as verified by either computed tomography (CT) scan or positron emission tomography (PET)-CT scan.

Patients who underwent CRS-HIPEC at our institution between April 2001 and September 2019 and were discharge from hospital were included in the study. Repeat CRS-HIPEC procedures of patients during the study period were excluded.

### CRS-HIPEC

We previously described how CRS-HIPEC was performed at our institution.^16^ In brief, cytoreduction was performed as described by Sugarbaker.^17^ An intraperitoneal chemotherapy agent appropriate for the patient’s malignancy type was prescribed by the medical oncologist and administered intraoperatively via a hyperthermia pump into a closed abdomen at 41–42 °C for 60 min. The Peritoneal Cancer Index (PCI)^17^ was used to document the extent of peritoneal disease, while the completeness of cytoreduction (CC) score^18^ was recorded to quantify the extent of cytoreduction.

### Postoperative Care

Following CRS-HIPEC, patients were typically monitored in the surgical intensive care unit (SICU) or high-dependency unit as deemed necessary by the primary surgeon and anesthetist. Postoperative complications were documented according to the Clavien–Dindo classification.^19^ Upon discharge, outpatient follow-up appointments were given at 1 week postdischarge, followed by a 1-month appointment, and thereafter 3-monthly appointments for 1 year, and 6-monthly appointments thereafter. Adjuvant chemotherapy was offered by medical oncologist as appropriate, and recurrences were documented.

### Key Definitions

Patients were categorized into three readmission categories:

1. Early readmission (ERA) was defined as the first unplanned (i.e., emergency, nonelective) hospitalization within 30 days (inclusive) post discharge from index CRS/HIPEC.
2. Late readmission (LRA) was defined as hospitalization occurring from 31 to 90 days after discharge from index CRS/HIPEC.
3. No readmission (NRA) was defined as no readmission within 90 days after discharge from index CRS-HIPEC.

Disease-free survival (DFS) was defined as duration between CRS-HIPEC and first recurrence or death from any cause, whichever occurred first, while overall survival (OS) was defined as duration between CRS-HIPEC and death from any cause.

Patients who did not experience the stated events for DFS and OS were censored at their last follow-up date.

### Statistics

For analysis of time to ERA and time to LRA, NRA patients were censored at day 90 post discharge from index CRS-HIPEC. For analysis of time to ERA, LRA and NRA patients were censored at day 30 post discharge from index CRS-HIPEC.

Patient demographic, oncological, operative, and postoperative factors were compared between ERA, LRA, and NRA using Fisher’s exact test and Kruskal–Wallis test for categorical and continuous variables, respectively. Cumulative incidence rate of RA was derived based on one minus the Kaplan–Meier estimate of the survival function for time to RA. Univariate and multivariable Cox proportional hazard (PH) regression models were used to examine the association of various factors with time to ERA and time to LRA. Variables with univariate *p* < 0.05 were included in the multivariable model. PH assumption was verified based on Schoenfeld residuals.

Follow-up duration was measured from discharge from CRS-HIPEC until date of last follow-up and estimated using the inverse Kaplan–Meier method. DFS and OS were estimated using Kaplan–Meier method. Differences in DFS and OS between patients in the three RA groups were compared using log-rank test.

Two-sided *p* value < 0.05 was considered statistically significant. All analyses were performed using SAS version 9.4 (SAS Institute Inc., Cary, NC).

## Results

Overall, 342 patients underwent CRS-HIPEC during the study duration. The demographics of the patients included in the study are summarized in Table 1. Our patient population had median age of 55 years, with ECOG status of 0 (87%) or 1 (12%). Colorectal cancer was the most common primary, accounting for nearly 40% of the cohort, followed by appendiceal cancer (25%), ovarian cancer (21%), and primary peritoneal disease (5%). Median PCI was 9, and after a median operative time of 495 min with median estimated blood loss of 1000 mL, CC-0 was achieved in 82% of cases, while CC-1 was achieved in 11%. Median length of hospitalization for CRS-HIPEC was 11 days (Table 2).

**Table 1.** Demographics, clinical, and treatment characteristics

	Total (*N* = 342)	NRA (*n* = 259)	ERA (*n* = 60)	LRA (*n* = 23)	*p* value
*Demographic*					
Age at CRS-HIPEC (years)	55 (14–79)	54 (22–79)	56 (25–76)	57 (14–74)	0.677
Gender					
Female	237 (69.3)	179 (69.1)	44 (73.3)	14 (60.9)	0.537
Male	105 (30.7)	80 (30.9)	16 (26.7)	9 (39.1)	
Ethnicity					
Chinese	256 (74.9)	187 (72.2)	49 (81.7)	20 (87.0)	0.482
Malay	23 (6.7)	17 (6.6)	5 (8.3)	1 (4.3)	
Indian	16 (4.7)	15 (5.8)	1 (1.7)	0 (–)	
Other	47 (13.7)	40 (15.4)	5 (8.3)	2 (8.7)	
*Clinical*					
ECOG performance status					
0	296 (86.5)	224 (86.5)	53 (88.3)	19 (82.6)	0.782
1	41 (12.0)	30 (11.6)	7 (11.7)	4 (17.4)	
Missing	5 (1.5)	5 (1.9)	0 (–)	0 (–)	
Comorbidities					
Absent	124 (36.3)	97 (37.5)	20 (33.3)	7 (30.4)	0.766
Present	218 (63.7)	162 (62.5)	40 (66.7)	16 (69.6)	
Type of comorbidities					
Hypertension	95 (27.8)	64 (24.7)	21 (35.0)	10 (43.5)	0.056
Diabetes	42 (12.3)	30 (11.6)	10 (16.7)	2 (8.7)	0.514
Hyperlipidemia	68 (19.9)	48 (18.5)	16 (26.7)	4 (17.4)	0.341
Ischemic heart disease	9 (2.6)	7 (2.7)	2 (3.3)	0 (–)	0.827
COPD	2 (0.6)	2 (0.8)	0 (–)	0 (–)	1.000
Asthma	7 (2.0)	3 (1.2)	3 (5.0)	1 (4.3)	0.065
Other malignancy	23 (6.7)	16 (6.2)	5 (8.3)	2 (8.7)	0.668
Others	132 (38.6)	105 (40.5)	21 (35.0)	6 (26.1)	0.334
Primary tumor site					
Colorectal^a^	129 (37.7)	99 (38.2)	19 (31.7)	11 (47.8)	0.052
Ovarian^b^	73 (21.3)	52 (20.1)	17 (28.3)	4 (17.4)	
Peritoneal	18 (5.3)	11 (4.2)	6 (10.0)	1 (4.3)	
Appendix	87 (25.4)	73 (28.2)	12 (20.0)	2 (8.7)	
Mesothelioma	13 (3.8)	7 (2.7)	4 (6.7)	2 (8.7)	
Others	22 (6.4)	17 (6.6)	2 (3.3)	3 (13.0)	
PCI score	9 (0–39)	8 (0–39)	13 (0–36)	14 (0–31)	0.088
No. of patients with nonmissing data	310	235	54	21	
Ascites					
Absent	200 (58.5)	161 (62.2)	29 (48.3)	10 (43.5)	0.113
Present	94 (27.5)	63 (24.3)	21 (35.0)	10 (43.5)	
Missing	48 (14.0)	35 (13.5)	10 (16.7)	3 (13.0)	
*Treatment*					
CRS procedure					
Subdiaphragmatic stripping	125 (36.5)	88 (34.0)	27 (45.0)	10 (43.5)	0.201
Gastrectomy	19 (5.6)	15 (5.8)	4 (6.7)	0 (–)	0.626
Colectomy	110 (32.2)	79 (30.5)	24 (40.0)	7 (30.4)	0.370
Small bowel resection	62 (18.1)	42 (16.2)	15 (25.0)	5 (21.7)	0.232
Splenectomy	58 (17.0)	42 (16.2)	14 (23.3)	2 (8.7)	0.274
THBSO	49 (14.3)	33 (12.7)	11 (18.3)	5 (21.7)	0.268
Cholecystectomy	57 (16.7)	44 (17.0)	11 (18.3)	2 (8.7)	0.618
Bladder resection	9 (2.6)	5 (1.9)	4 (6.7)	0 (–)	0.131
Other procedure(s)	100 (29.2)	77 (29.7)	15 (25.0)	8 (34.8)	0.623
HIPEC agent					
Cisplatin	109 (31.9)	74 (28.6)	25 (41.7)	10 (43.5)	0.090
Mitomycin C	215 (62.9)	172 (66.4)	32 (53.3)	11 (47.8)	
Others^c^	12 (3.5)	7 (2.7)	3 (5.0)	2 (8.7)	
Missing	6 (1.8)	6 (2.3)	0 (–)	0 (–)	
Duration of CRS-HIPEC, mins	495 (245–1070)	475 (245–1070)	585 (285–1020)	500 (310–795)	0.016
No. of patients with nonmissing data:	297	221	54	22	
CC score					
0	279 (81.6)	212 (81.9)	46 (76.7)	21 (91.3)	0.155
1	36 (10.5)	26 (10.0)	10 (16.7)	0 (–)	
2	4 (1.2)	3 (1.2)	1 (1.7)	0 (–)	
3	1 (0.3)	0 (–)	1 (1.7)	0 (–)	
Missing	22 (6.4)	18 (6.9)	2 (3.3)	2 (8.7)	
Chest tube placement					
No	154 (45.0)	126 (48.6)	20 (33.3)	8 (34.8)	0.096
Yes	175 (51.2)	122 (47.1)	39 (65.0)	14 (60.9)	
Missing	13 (3.8)	11 (4.2)	1 (1.7)	1 (4.3)	
Stoma creation					
No	238 (69.6)	190 (73.4)	36 (60.0)	12 (52.2)	0.020
Yes	104 (30.4)	69 (26.6)	24 (40.0)	11 (47.8)	
Estimated blood loss (ml)	1000 (0–11,000)	900 (0–11,000)	1000 (200–5100)	800 (0–3500)	0.439
No. of patients with nonmissing data:	327	248	58	21	
Intraoperative blood transfusions					
No	110 (32.2)	90 (34.7)	13 (21.7)	7 (30.4)	0.236
Yes	224 (65.5)	163 (62.9)	46 (76.7)	15 (65.2)	
Missing	8 (2.3)	6 (2.3)	1 (1.7)	1 (4.3)	

*NRA* No readmission, *ERA* Early readmission, *LRA* Late readmission, *CRS* Cytoreduction surgery, *HIPEC* Hyperthermic intraperitoneal chemotherapy, *ECOG* Eastern Cooperative Oncology Group, *COPD* Chronic obstructive pulmonary disease, *PCI* Peritoneal cancer index, *THBSO* Total abdominal hysterectomy with bilateral salpingo-oophorectomy, *CC* Completeness of cytoreduction

Data presented as median (range) if variable is continuous, and number (%) if variable is categorical

*p* value based on Kruskal–Wallis test for continuous variable and Fisher’s exact test for categorical variable

^a^Included one patient who had an additional primary tumor in endometrium

^b^Included one patient who had an additional primary gastric tumor

^c^Included doxorubicin, oxaliplatin, and fluorouracil

**Table 2.** Postoperative characteristics and recurrence

	Total (*N* = 342)	NRA (*n* = 259)	ERA (*n* = 60)	LRA (*n* = 23)	*p* value
Postoperative complications					
No	145 (42.4)	122 (47.1)	15 (25.0)	8 (34.8)	0.006
Yes	197 (57.6)	137 (52.9)	45 (75.0)	15 (65.2)	
Worst grade of postoperative complications					
No complication	145 (42.4)	122 (47.1)	15 (25.0)	8 (34.8)	< 0.001
G1	48 (14.0)	37 (14.3)	7 (11.7)	4 (17.4)	
G2	92 (26.9)	69 (26.6)	17 (28.3)	6 (26.1)	
G3	44 (12.9)	20 (7.7)	19 (31.7)	5 (21.7)	
G4	13 (3.8)	11 (4.2)	2 (3.3)	0 (–)	
Length of SICU stay (days)	0 (0–40)	0 (0–40)	1 (0–5)	1 (0–3)	0.015
No. of patients with nonmissing data	341	258	60	23	
Length of hospital stay (days)	11 (5–141)	11 (5–141)	14 (7–66)	13 (8–86)	0.001
No. of recurred patients	163	116	32	15	–
Site of relapse among recurred patients:					
Peritoneum	110 (67.5)	82 (70.7)	18 (56.3)	10 (66.7)	0.293
Lymph nodes	36 (22.1)	22 (19.0)	11 (34.4)	3 (20.0)	0.181
Lung	38 (23.3)	29 (25.0)	5 (15.6)	4 (26.7)	0.490
Liver	36 (22.1)	22 (19.0)	9 (28.1)	5 (33.3)	0.249
Bone	7 (4.3)	4 (3.4)	0 (–)	3 (20.0)	0.016
Skin	0 (–)	0 (–)	0 (–)	0 (–)	–
Others	40 (24.5)	28 (24.1)	5 (15.6)	7 (46.7)	0.083

*NRA* No readmission, *ERA* Early readmission, *LRA* Late readmission, *SICU* Surgical intensive care unit

Data presented as median (range) if variable is continuous, and number (%) if variable is categorical

*p* value based on Kruskal–Wallis test for continuous variable and Fisher’s exact test for categorical variable

Sixty patients had ERA, 23 had LRA, and 259 had NRA within 90 days post discharge from CRS-HIPEC. Median time to ERA and LRA was 8 and 51 days, respectively.

Median age between RA groups was comparable, as was the distribution of ECOG status. Ovarian (ERA 28% versus LRA 17%), appendiceal (ERA 20% versus LRA 9%), and peritoneal (ERA 10% versus LRA 4.3%) primaries were more common in ERA group, while colorectal primary was more common is LRA group (LRA 48% versus ERA 32%), but these differences in distribution did not reach statistical significance. Median PCI score was comparable between RA groups, and CC-0 score was achieved in 77% of LRA group and 91% of ERA group (*p* = 0.155). The most common cytoreductive procedure performed across both readmission groups was subdiaphragmatic stripping, followed by colectomy and small bowel resection.

### Causes of Readmission Following CRS-HIPEC

The majority (46%) of RA were due to gastrointestinal complaints, such as abdominal pain, bloatedness, nausea, and vomiting (Table 3). A total of 15% of RA were a result of stoma-related complications (e.g., high stoma output), and 8% from superficial wound infections. Although there was a higher percentage of LRA patients (26%) with stoma-related readmission compared with ERA patients (10%), on balance reasons for RA were similar between these two groups (*p* = 0.450).

**Table 3.** Readmission characteristics

	Total (*N* = 83)	ERA (*n* = 60)	LRA (*n* = 23)	*p* value
*Readmission reason*				
GI symptoms	38 (45.8)	29 (48.3)	9 (39.1)	0.450
Wound infection	7 (8.4)	5 (8.3)	2 (8.7)	
Other infection	4 (4.8)	3 (5.0)	1 (4.3)	
Stoma related	12 (14.5)	6 (10.0)	6 (26.1)	
Others	22 (26.5)	17 (28.3)	5 (21.7)	
*Death within 30 days of readmission*				
Excluding alive patients with < 30 days follow-up:	82	60	22	
No	81 (98.8)	59 (98.3)	22 (100)	1.000
Yes	1 (1.2)	1 (1.7)	0 (–)	

*ERA* Early readmission, *LRA* Late readmission

Data presented as number (%)

*p* value based on Fisher’s exact test

### Factors Affecting Readmissions Following CRS-HIPEC

Comparison of operative and postoperative variables showed that, compared with the NRA and LRA groups, patients with ERA had the longest duration of CRS-HIPEC [median 475 min (NRA) versus 500 min (LRA) versus 585 min (ERA); *p* = 0.016], highest percentage with grade III–IV postoperative complication [12% (NRA) versus 22% (LRA) versus 35% (ERA); *p* < 0.001] and longest index hospital admission for CRS-HIPEC [median 11 days (NRA) versus 13 days (LRA) versus 14 days (ERA); *p* = 0.001] (Tables 1 and 2).

There were significantly more patients who had stoma created at index CRS-HIPEC among LRA group (48%) than ERA (40%) or NRA (27%) (*p* = 0.020). Demographic and oncological variables showed no significant difference between the three readmission groups (Table 1).

Univariate Cox regression analysis identified eight significant predictors for ERA: PCI score, intraoperative bladder resection, intraoperative chest tube placement, duration of CRS-HIPEC, CC score, intraoperative blood transfusion, duration of hospital stay, and high-grade postoperative complication (grade III–IV). On multivariate analysis, only high-grade postoperative complication continued to be significant (HR 3.64, 95% CI 1.47–9.02; relative to no complications) (Table 4). For LRA, the variables significant on univariate analysis, which also remained significant on multivariate analysis, were presence of hypertension (HR 2.71, 95% CI 1.17–6.28) and stoma creation (HR 2.83, 95% CI 1.23–6.50) (Table 5).

**Table 4.** Predictors of early readmission

	Univariate Cox		Multivariable Cox	
	HR (95% CI)	*p* value	HR (95% CI)	*p* value
Age at CRS-HIPEC (per year increase)	1.01 (0.98–1.03)	0.612		
Gender: male versus female	0.79 (0.45–1.40)	0.424		
Ethnicity: Malay versus Chinese	1.32 (0.53–3.31)	0.554		
Ethnicity: Indian versus Chinese	0.39 (0.05–2.81)	0.350		
Ethnicity: others versus Chinese	0.54 (0.21–1.35)	0.184		
ECOG performance status: 1 versus 0	1.00 (0.46–2.20)	0.998		
Comorbidities: absent versus present	0.83 (0.48–1.42)	0.489		
Hypertension: yes versus no	1.48 (0.87–2.52)	0.148		
Diabetes: yes versus no	1.61 (0.82–3.17)	0.171		
Hyperlipidemia: yes versus no	1.47 (0.83–2.61)	0.186		
Ischemic heart disease: yes versus no	1.29 (0.31–5.27)	0.726		
COPD: yes versus no	UD	0.985		
Asthma: yes versus no	3.07 (0.96–9.81)	0.058		
Other malignancy: yes versus no	1.42 (0.57–3.54)	0.456		
Other comorbidities: yes versus no	0.86 (0.50–1.45)	0.563		
Tumor site: ovarian versus colorectal	1.67 (0.87–3.21)	0.125		
Tumor site: peritoneal versus colorectal	2.38 (0.95–5.95)	0.065		
Tumor site: appendix versus colorectal	0.98 (0.48–2.02)	0.952		
Tumor site: mesothelioma versus colorectal	2.44 (0.83–7.19)	0.105		
Tumor site: others versus colorectal	0.58 (0.14–2.49)	0.464		
PCI score (per unit increase)	1.03 (1.00–1.06)	0.038	0.98 (0.94–1.03)	0.446
Had ascites: yes versus no	1.64 (0.93–2.87)	0.086		
Subdiaphragmatic stripping: yes versus no	1.49 (0.89–2.47)	0.128		
Gastrectomy: yes versus no	1.21 (0.44–3.34)	0.712		
Colectomy: yes versus no	1.52 (0.91–2.55)^a^	0.111		
Small bowel resection: yes versus no	1.56 (0.87–2.80)	0.136		
Splenectomy: yes versus no	1.53 (0.84–2.78)	0.166		
THBSO: yes versus no	1.47 (0.77–2.83)	0.247		
Cholecystectomy: yes versus no	1.17 (0.61–2.25)	0.638		
Bladder resection: yes versus no	2.99 (1.08–8.24)	0.035	Note^b^	
Other CRS procedure(s): yes versus no	0.77 (0.43–1.39)	0.389		
HIPEC agent: mitomycin C versus cisplatin	0.64 (0.38–1.08)	0.093		
HIPEC agent: others versus cisplatin	1.14 (0.34–3.77)	0.832		
Duration of CRS-HIPEC (per 10min increase)	1.02 (1.01–1.04)	0.007	1.01 (0.98–1.04)	0.528
CC score: ≥1 versus 0	1.99 (1.06–3.76)	0.034	1.49 (0.59–3.80)	0.401
Chest tube placement: yes versus no	1.88 (1.10–3.22)	0.022	1.12 (0.53–2.38)	0.767
Stoma creation: yes versus no	1.65 (0.99–2.77)	0.056		
Blood loss (per 100-ml increase)	1.01 (0.99–1.03)	0.425		
Intraoperative blood transfusion: yes versus no	1.86 (1.00–3.44)	0.049	1.36 (0.61–3.06)	0.450
Postoperative complication: G1–G2 versus none	1.72 (0.90–3.29)	0.098	1.86 (0.89–3.89)	0.098
Postoperative complication: G3–G4 versus none	4.49 (2.31–8.71)	< 0.001	3.64 (1.47–9.02)	0.005
Length of SICU stay (per day increase)	1.00 (0.94–1.08)	0.917		
Length of hospital stay (per day increase)	1.01 (1.00–1.03)	0.028	1.00 (0.97–1.02)	0.758

*HR* Hazard ratio, *CI* Confidence interval, *CRS* Cytoreduction surgery, *HIPEC* Hyperthermic intraperitoneal chemotherapy, *ECOG* Eastern Cooperative Oncology Group, *COPD* Chronic obstructive pulmonary disease, *PCI* Peritoneal cancer index, *THBSO* Total abdominal hysterectomy with bilateral salpingo-oophorectomy, *CC* Completeness of cytoreduction, *SICU* Surgical intensive care unit, *UD* No event in one of the categories

*p* value based on Wald’s test

^a^Violated PH assumption

^b^Excluded from model due to small sample size

**Table 5.** Predictors of late readmission

	Univariate Cox		Multivariable Cox	
	HR (95% CI)	*p* value	HR (95% CI)	*p* value
Age at CRS-HIPEC (per year increase)	1.00 (0.97–1.04)	0.888		
Gender: male versus female	1.34 (0.58–3.08)	0.499		
Ethnicity: Malay versus Chinese	0.66 (0.09–4.92)	0.686		
Ethnicity: Indian versus Chinese	UD	0.990		
Ethnicity: others versus Chinese	0.46 (0.11–1.98)	0.299		
ECOG performance status: 1 versus 0	1.85 (0.63–5.44)	0.263		
Comorbidities: absent versus present	0.69 (0.28–1.68)	0.415		
Hypertension: yes versus no	2.28 (1.00–5.21)	0.050	2.71 (1.17–6.28)	0.020
Diabetes: yes versus no	0.81 (0.19–3.47)	0.779		
Hyperlipidemia: yes versus no	0.90 (0.31–2.64)	0.843		
Ischemic heart disease: yes versus no	UD	0.992		
COPD: yes versus no	UD	0.990		
Asthma: yes versus no	4.60 (0.62–34.12)	0.136		
Other malignancy: yes versus no	1.46 (0.34–6.22)	0.610		
Other comorbidities: yes versus no	0.53 (0.21–1.36)	0.187		
Tumor site: ovarian versus colorectal	0.76 (0.24–2.39)	0.638		
Tumor site: peritoneal versus colorectal	0.81 (0.11–6.29)	0.841		
Tumor site: appendix versus colorectal	0.26 (0.06–1.17)	0.079		
Tumor site: mesothelioma versus colorectal	2.24 (0.50–10.10)	0.295		
Tumor site: others versus colorectal	1.43 (0.40–5.11)	0.587		
PCI score (per unit increase)	1.01 (0.97–1.06)	0.646		
Had ascites: yes versus no	2.36 (0.98–5.66)^a^	0.055		
Subdiaphragmatic stripping: yes versus no	1.41 (0.62–3.21)	0.419		
Gastrectomy: yes versus no	UD	0.988		
Colectomy: yes versus no	0.99 (0.41–2.41)	0.988		
Small bowel resection: yes versus no	1.38 (0.51–3.71)	0.528		
Splenectomy: yes versus no	0.45 (0.11–1.91)	0.278		
THBSO: yes versus no	1.86 (0.69–5.00)	0.222		
Cholecystectomy: yes versus no	0.46 (0.11–1.96)	0.294		
Bladder resection: yes versus no	UD	0.989		
Other CRS procedure(s): yes versus no	1.17 (0.50–2.76)	0.721		
HIPEC agent: mitomycin C versus cisplatin	0.48 (0.20–1.13)	0.093		
HIPEC agent: others versus cisplatin	1.67 (0.37–7.62)	0.508		
Duration of CRS-HIPEC (per 10-min increase)	1.01 (0.98–1.04)	0.469		
CC score: ≥ 1 versus 0	UD	0.990		
Chest tube placement: yes versus no	1.81 (0.76–4.32)	0.181		
Stoma creation: yes versus no	2.41 (1.06–5.46)	0.035	2.83 (1.23–6.50)	0.015
Blood loss (per 100-ml increase)	0.99 (0.95–1.04)	0.730		
Intraoperative blood transfusion: yes versus no	1.16 (0.47–2.85)	0.743		
Postoperative complication: G1–G2 versus none	1.38 (0.54–3.49)	0.500		
Postoperative complication: G3–G4 versus none	2.42 (0.79–7.39)	0.122		
Length of SICU stay (per day increase)	0.96 (0.79–1.15)	0.631		
Length of hospital stay (per day increase)	1.01 (0.99–1.03)	0.194		

*HR* hazard ratio, *CI* confidence interval, *CRS* cytoreduction surgery, *HIPEC* hyperthermic intraperitoneal chemotherapy, *ECOG* Eastern Cooperative Oncology Group, *COPD* chronic obstructive pulmonary disease, *PCI* peritoneal cancer index, *THBSO* total abdominal hysterectomy with bilateral salpingo-oophorectomy, *CC* completeness of cytoreduction, *SICU* surgical intensive care unit, *UD* no event in one of the categories

*p* value based on Wald’s test

^a^Violated PH assumption

### Relationship Between Readmission and Survival Outcomes

The median follow-up of NRA, ERA, and LRA groups was 2.0, 4.5, and 1.9 years, respectively.

### Disease-Free Survival

**Fig. 1.**
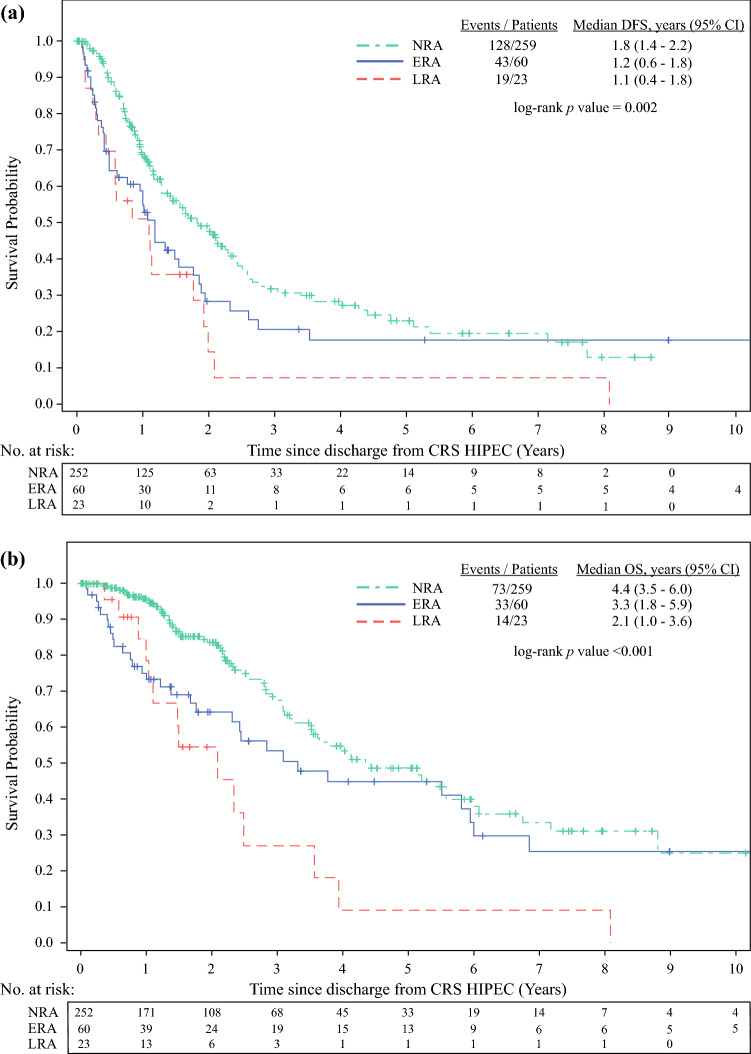
Kaplan–Meier curves of **a** DFS and **b** OS stratified by ERA, LRA, and NRA

Patients with readmission had significantly worse DFS than NRA patients (Fig. 1). Median DFS was 1.1 years (95% CI 0.4–1.8 years) for LRA patients and 1.2 years (95% CI 0.6–1.8 years) for ERA patients, both being lower than the corresponding 1.8 years (95% CI 1.4–2.2 years) for NRA patients (*p* = 0.002; Table 6, Fig. 1a).

**Table 6.** Survival outcomes

	Total (*N* = 342)	NRA (*n* = 259)	ERA (*n* = 60)	LRA (*n* = 23)	*p* value*
*Follow-up duration (years)*					
Median (95% CI)	2.1 (1.7–2.3)	2.0 (1.5–2.2)	4.5 (1.8–9.0)	1.9 (0.8–NE)	0.003
*Disease-free survival (DFS)*					
No. of recurrences/deaths	190	128	43	19	0.002
Median DFS, years (95% CI)	1.6 (1.3–1.9)	1.8 (1.4–2.2)	1.2 (0.6–1.8)	1.1 (0.4–1.8)	
6-Month DFS, % (95% CI)	82.7 (78.0–86.5)	88.8 (83.9–92.3)	64.0 (50.3–74.8)	69.6 (46.6–84.2)	
1-Year DFS, % (95% CI)	65.5 (59.7–70.7)	68.7 (61.9–74.6)	58.4 (44.6–69.9)	50.6 (28.6–69.0)	
2-Year DFS, % (95% CI)	41.4 (35.1–47.7)	48.1 (40.4–55.3)	28.0 (16.2–41.1)	**14.2 (2.6–35.1)**	
*Overall survival (OS)*					
No. of deaths	120	73	33	14	< 0.001
Median OS, years (95% CI)	3.9 (3.2–5.4)	4.4 (3.5–6.0)	3.3 (1.8–5.9)	2.1 (1.0–3.6)	
1-Year OS, % (95% CI)	90.7 (86.7–93.5)	95.8 (92.0–97.8)	75.1 (61.6–84.5)	78.6 (52.0–91.5)	
2-Year OS, % (95% CI)	77.8 (71.9–82.6)	83.6 (77.0–88.5)	64.1 (49.4–75.6)	54.4 (28.8–74.2)	

*NE* not estimable

*Based on log-rank test

Numbers in bold based on small no. of patients at risk

### Overall Survival

LRA patients had worst median OS (2.1 years, 95% CI 1.0–3.6 years), followed by ERA patients (3.3 years, 95% CI 1.8–5.9 years) and NRA patients (4.4 years, 95% CI 3.5–6.0 years) (*p* < 0.001; Table 6; Fig. 1b).

## Discussion

Internationally, reported rates of postoperative readmission after CRS-HIPEC range from 14.8 to 15.9% for ERA and from 3.9 to 11% for LRA.^11^,^15^,^20^ Known predictors include older age, number of previous surgical procedures, postoperative complications, and length of index hospitalization.^10^ Lee et al. went on to the compare the differences in predictors for RA at 30 versus 31–90 days. ECOG of 3 or more, intraoperative splenectomy, low anterior resection, partial colectomy, and stoma creation were independent predictors of 30-day RA, while gastric tumor, operative time, intraoperative low anterior resection or partial colectomy, and stoma creation were predictors for 31–90-day RA.^11^ Beyond 90 days, age and intraoperative colonic resection have been reported as the only independent risk factors for 6-month readmission.^21^ In our study cohort, we found similar rates of early and late readmission of 18.5% and 7.4%, respectively, with a majority of RA occurring within 2 weeks post discharge. Only high-grade postoperative complications, stoma creation, and hypertension predicted ERA and LRA. In our study population, 57 patients (16.7%) had high-grade complications, and 21 of these patients had ERA. In contrast, 176 patients (51.5%) had risk factors for LRA, including those with both hypertension and stoma creation, hypertension only, and stoma creation only. Compared with patients with no LRA risk factors, risk of LRA was highest amongst patients with both hypertension and stoma creation (HR = 7.67, *n* = 23 [6.7%]), followed by patients with stoma creation only [HR = 2.83, *n* = 81 (23.7%)] and patients with hypertension only [HR = 2.71, *n* = 72 (21.1%)], with our Cox model suggesting that these two risk factors have a multiplicative effect in predisposing patients to late readmission.

Postoperative readmission has been reported to be related to adverse survival outcomes in patients who had undergone surgery for cancers of various organs, such as brain, pancreas, esophagus, and stomach.^22^–^25^ Proposed contributors to this correlation include postoperative complications, infection, and metastatic disease.^24^,^25^ Others have also reported that readmission within 30 days of surgery is associated with delay in postoperative chemotherapy, which in turn is associated with poorer DFS and OS.^26^ In the context of post-pancreatic-cancer surgery, Reddy et al. reported that, compared with NRA, 0–30-day readmissions had lower median OS but comparable 5-year survival. Meanwhile 30–365-day readmissions had both lower median as well as 5-year survival compared with those without 30–365-day readmission.^23^ These findings suggested that, if the patients who required ERA survive the first few years following the index operation, their long-term outcome is comparable to those who had no readmission, whereas patients who required LRA have worse long-term outcomes regardless. We found that ERA and LRA patients had comparable median DFS, though significantly lower than NRA. Overall survival was worst amongst LRA, followed by ERA and NRA.

To the best of the authors’ knowledge, this study is the first to compare survival outcomes in ERA, LRA, and NRA patients post CRS-HIPEC. Unfortunately, for our study, the median follow-up duration was not long enough to comment on 5-year survival outcomes. However, the survival curves at the 5-year mark seem to resonate the survival patterns reported by Reddy et al. The exact reason for these patterns of survival outcomes lies beyond the scope of this study. However, in broad conceptual terms, it may be reasonable to speculate that, post CRS-HIPEC, ERA is associated with potentially significant yet reversible causes, while LRA involves both significant and irreversible pathologies.

The adverse effect of postoperative morbidity on survival outcomes has been reported in literature for both oncological and nononcological surgeries.^27^–^29^ In the past, our center has also reported the association of high-grade complications with poor OS among post CRS-HIPEC patients; the 5-year OS rate of patients who experienced no postoperative, low-grade, and high-grade complications was found to be 52.8%, 37.0%, and 43.0%, respectively.^30^ As the results of the current study also show that high-grade morbidity is the sole independent risk factor for early readmission, it would be reasonable to infer that postoperative morbidity contributes to the association of ERA with poor survival outcomes.

Up to 30% of our CRS-HIPEC patients required stoma creation, a majority of which were defunctioning ileostomies for colorectal resection. Furthermore, intraoperative stoma creation was found to be a significant predictor of LRA, which was in turn associated with poorer survival outcomes. In patients with primary colorectal malignancies without peritoneal disease, post-stoma readmissions are known to be common and often occur within 30 days of discharge.^31^–^33^ Common early complications after stoma creation include skin irritation, pain, stoma retraction, and necrosis, while later in the clinical course, stoma patients may experience parastomal hernia, prolapse, stenosis, high output, and nutritional deficiencies.^34^,^35^ Our analysis of stoma formation in the setting of CRS-HIPEC found there was a greater percentage of stoma patients among the LRA than ERA group, from which we can cautiously infer that, for post CRS-HIPEC readmissions, stoma creation and its late complications may play a greater role in predisposing a patient to LRA. A small subgroup analysis of the 12 patients who were readmitted for stoma-related reasons showed that the vast majority of ERA was due to high stoma output (83%), while for LRA only 50% presented with high stoma output and complications related to stoma reversal accounted for a sizable proportion (33%). However, these figures are limited by small sample size, and a detailed analysis of stoma type, timing of reversal, related complications, and their association with readmissions and survival requires further investigation.

Based on the association of unplanned readmission with adverse survival outcomes seen in our results, future studies should investigate interventions that may reduce readmission and hence improve survival. One such intervention aimed to reduce LRA may include vigilant patient education and follow-up for those who have stoma creation during CRS-HIPEC, with special attention to known late complications such as parastomal herniations or post reversal strictures, infections, or anastomotic leak/breakdown. As our study also found that nearly half of all readmissions occurred within the first 15 days post discharge, with the majority presenting with gastrointestinal symptoms, it would be prudent to investigate whether early postoperative intervention with comprehensive discharge planning that includes appropriate discharge advice, streamlined wound care, dietician review, and close follow-up within the first month may help to off-load the high rates of ERA and provide survival benefits.

The retrospective design and relatively small number in this study may have resulted in selection bias and failure to elucidate other factors that may contribute to the differences in survival outcomes seen in the respective readmission groups. Longer follow-up may be required to further identify factors affecting long-term survival outcomes in our study population.

## Conclusions

In this study conducted at the largest CRS-HIPEC center in Southeast Asia, unplanned postoperative readmission occurred at a rate of 18.5% for 30 days post discharge and 7.4% for 31–90 days. Unique sets of independent predictors were identified for these two readmission types: high-grade postoperative complication was a predictor of ERA, while stoma creation and hypertension were predictors for LRA. In addition, there were worse survival outcomes for patients with LRA as compared with ERA and NRA. Future studies may need to explore the association of poor survival with readmissions, to better identify effective measures to minimize unplanned hospitalizations post CRS-HIPEC.
